# SFAs do not impair endothelial function and arterial stiffness^1^,^2^,^3^

**DOI:** 10.3945/ajcn.113.063644

**Published:** 2013-08-14

**Authors:** Thomas AB Sanders, Fiona J Lewis, Louise M Goff, Philip J Chowienczyk

**Affiliations:** 1 From the Diabetes and Nutritional Sciences Division (TABS, FJL, and LMG) and British Heart Foundation Centre (PJC), King's College London, London, United Kingdom.

## Abstract

**Background:** It is uncertain whether saturated fatty acids (SFAs) impair endothelial function and contribute to arterial stiffening.

**Objective:** We tested the effects of replacing SFAs with monounsaturated fatty acids (MUFAs) or carbohydrates on endothelial function and arterial stiffness.

**Design:** With the use of a parallel-designed randomized controlled trial in 121 insulin-resistant men and women, we measured vascular function after 1 mo of consumption of a high-SFA (HS) diet and after 24 wk after random assignment to the HS diet or diets that contained <10% SFAs and were high in either MUFAs or carbohydrates. The primary outcome was a change in flow-mediated dilation (FMD), and secondary outcomes were changes in carotid to femoral pulse wave velocity (PWV) and plasma 8-isoprostane F_2α_-III concentrations.

**Results:** For 112 participants with data available for analysis on the specified outcomes, no significant differences were shown. FMD with the HS reference diet was 6.7 ± 2.2%, and changes (95% CIs) after 6 mo of intervention were +0.3 (−0.4, 1.1), −0.2 (−0.8, 0.5), and −0.1 (−0.6, 0.7) with HS, high-MUFA (HM), and high-carbohydrate (HC) diets, respectively. After consumption of the HS reference diet, the geometric mean (±SD) PWV was 7.67 ± 1.62 m/s, and mean percentages of changes (95% CIs) were −1.0 (−6.2, 4.3) with the HS diet, 2.7 (−1.4, 6.9) with the HM diet, and −1.0 (−5.5, 3.4) with the HC diet. With the HS reference diet, the geometric mean (±SD) plasma 8-isoprostane F_2α_-III concentration was 176 ± 85 pmol/L, and mean percentage of changes (95% CIs) were 1 (−12, 14) with the HS diet, 6 (−5, 16) with the HM diet, and 4 (−7, 16) with the HC diet.

**Conclusion:** The replacement of SFAs with MUFAs or carbohydrates in healthy subjects does not affect vascular function. This trial was registered at Current Controlled Trials (http://www.controlled-trials.com/ISRCTN) as ISRCTN 29111298.

## INTRODUCTION

It has been well established that SFAs influence plasma lipid concentrations, but their effects on arterial function remain uncertain (1). Arteries stiffen with age contributing to increased cardiovascular disease mortality (2). The mechanisms that result in arterial ageing are uncertain but may involve an increased production of reactive oxygen species, which decrease the bioavailability of nitric oxide (NO)^4^, which is a molecule produced by the vascular endothelium that plays an important role in the regulation of vascular tone and function (3). Flow-mediated dilation (FMD) of the brachial artery (4) is a technique used to assess the capacity of the artery to dilate in response to NO synthesized by the endothelium in response to an increase in shear stress. Insulin resistance has been shown to be associated with impaired endothelial function (5), and there is a close correlation between insulin sensitivity and basal insulin production in healthy subjects (6). There is also abundant evidence of increased vascular superoxide production, decreased tissue glutathione, impaired endothelial-dependent relaxation, and increased NADPH oxidase activity leading to the uncoupling of endothelial NO synthase in diabetes (7). The plasma concentration of plasma 8-isoprostane F_2α_-III is regarded as a robust marker of increased free-radical oxidation, and we have previously shown that high-fat meals (50 g) compared with carbohydrates result in increased plasma concentrations of this metabolite (8, 9) and transiently impaired endothelial function (8, 10).

It is currently uncertain whether diets with a reduced SFA content influence endothelial function. Keogh et al (11) reported that FMD was impaired after a high-SFA (HS) diet compared with high-MUFA (HM), high-PUFA, or high-carbohydrate (HC) diets. Several studies have also reported that adherence to a Mediterranean style diet is associated with improvements in endothelial function (12–14), which have often been attributed to the consumption of virgin olive oil, which is rich in MUFAs. However, it has also been suggested that the effect on endothelial function is mediated by antioxidants in virgin olive oil (15). Hall (16) reviewed the effects of dietary fat modification on vascular function and concluded that the quantity and quality of the published evidence was insufficient to draw any firm conclusions regarding the effects of SFAs compared with MUFAs. The Reading, Imperial, Surrey, Cambridge, and Kings (17) was a multi-center randomized, controlled dietary intervention, in subjects at increased risk of metabolic syndrome, designed to assess the impact of modifying the type and level of fat on insulin sensitivity by replacing SFAs with either MUFAs or carbohydrates. We have previously reported that a reduction in SFA intake led to a fall in total and LDL cholesterol but did not influence insulin sensitivity (17). In the present study we report the results from a substudy that investigated the influence of saturated fat reduction on endothelial function and arterial stiffness.

## SUBJECTS AND METHODS

### Subjects

The Reading, Imperial, Surrey, Cambridge, and Kings study (http://www.controlled-trials.com/ISRCTN; ISRCTN 29111298), which was conducted between January 2004 and December 2007, was approved by the South East Multi-center Research Ethics Committee (reference MREC04/MRE01/2). Men and women aged 30–70 y were recruited from the general population by using an advertisement and were selected as being at risk of metabolic syndrome on the basis of measurements of waist circumference and blood pressure, fasting insulin, glucose, blood pressure, and lipid concentrations as described elsewhere (17). Informed written consent was obtained, and a small remuneration was given for participation in the study.

### Study design

Participants were randomly allocated to one of 5 diets by using a parallel design. In the current study, measurements of vascular function were only made with 3 diets with high–glycemic index carbohydrates in King's College London and Imperial College London cohorts. After a 1-mo run-in on a reference HS diet, participants were randomly assigned to the HS diet or diets that contained <10% energy in fat in which SFAs were replaced with MUFAs or carbohydrates for 24 wk. Participants were advised that the dietary advice was designed for weight maintenance.

The dietary exchange model was based on average UK food-intake data. It was estimated that approximately two-fifths of the sources of fat (yellow fat spreads, cooking oils, cheese, milk, biscuits, cakes, buns, and pastries) could be exchanged. The model used the removal of the exchangeable fat and replacement by study foods with a specific fatty acid profile. The model assumed milk intakes to be 230 and 190 mL/d and cheese intakes to be 17 and 12 g/d in men and women, respectively. The HS reference diet used full-fat milk (3.8 g/100 mL) and cheese (35 g/100 g), whereas these were replaced with skimmed milk (0.1 g fat/100 g) and half-fat cheese (18 g/100 g) in HM and HC diets. It was assumed that fat-spread intake would be 20 and 15 g/d in men and women, respectively. Spreads and vegetable oils were supplied by Unilever Bestfoods and contained <1%* trans* fatty acids. The amount of fat was 80 g/100 g in the HS reference and HM diets and 27.4 g/100 g in the HC diet. Ratios of SFA:MUFA:PUFA in the spreads were 50:26:24 in the HS reference diet, 18:60:23 in the HM diet, and 21:52:27 in the HC diet; the ratio of linoleic:linolenic acid in all spreads was 5:1. The dietary model assumed 11 and 6 g cooking oil/d would be used by men and women, respectively, in the SFA and MUFA diets, and 5 cooking oil/d would be used in the HC diet. Ratios of SFA:MUFA:PUFA were 51:39:11 in the HS reference diet, 14:75:11 in the HM diet, and 10:57:33 in the HC diet. Participants were provided with a standard salad dressing or HM or reduced-fat versions. The vitamin E content was standardized to 37.5 mg/100 g in spreads and salad dressings and to 62 mg/100 g in oils. To compensate for the fat provided by dairy foods, snack foods with a HM content were used (nuts and potato crisps). With the HC diet, participants were advised to consume additional portions of bread, potatoes, and rice to compensate for the energy reduction that resulted from decreased fat intake (*see* Online Supplemental Material under “Supplemental data” in the online issue for details of the advice). The dietary intervention has been described in detail elsewhere (18).

Power calculations were based on a mean FMD of 6.2% with a within-subject SD for measurement on different days of 1.1%; these calculated suggested that 32 subjects/group completing the study would give 90% power to detect a 1% difference in FMD at *P* = 0.01, which would permit the detection a 1% difference between groups at *P* = 0.05 when multiple comparisons were allowed for. With the allowance of a 15% dropout rate, we aimed to recruit 38 subjects/group. Prespecified secondary outcomes were changes in arterial stiffness measured as carotid to femoral pulse wave velocity (PWV_c-f_) and changes in plasma isoprostane concentrations. All other outcomes were exploratory. Random assignment to treatments used a minimization procedure to balance by age, sex, waist circumference, and HDL cholesterol and was completed by using a computer program.

### Methods

Participants were advised before each visit for the measurement of vascular function to avoid strenuous physical activity, foods high in fat, caffeine, or alcohol on the previous day; subjects were provided with a list of foods to avoid and a low-fat evening meal (<10 g fat; 2–3 MJ) before fasting overnight from 2200. The following morning, participants attended a clinic in the fasting state between 0800 and 1100. Measurements of weight, waist circumference, body composition, and seated blood pressure were made, and an indwelling venous cannula was inserted into the forearm. Fasting blood samples were collected. Blood for isoprostane analysis was collected into a chilled 5-mL monovette (Sarstedt) that contained trisodium citrate. The cyclooxygenase inhibitor indomethacin solution [38 μL of 2 mmol/L aqueous NaHCO_3_ (50 g/L)] was added to give a final concentration of 15 μmol indomethacin/L. The sample was allowed to stand on ice at 4°C for 30 min and centrifuged at 2400 × *g* for 15 min. Plasma was collected, and antioxidant was added, before freezing and storage at −70°C; 4 μL of 5-mmol butylated hydroxytoluene/L ethanol was added to each milliliter of plasma to give a final concentration of 20 μmol/L. 8-Isoprostane F2α-III [9α,-11α,-15S-trihydroxy-(8β)-prosta-5Z,13E-dien-1-oic acid)] was determined by using isotope-dilution mass spectroscopy with iso-8-prostaglandin F 2α-III-17,18,19,20–D4 (Cayman Chemical Co) as an internal standard as previously described (19). To increase the precision of the analysis, pairs of samples from the same participant were analyzed in the same run; between- and within-run interassay variabilities were 4% and 7%, respectively.

After the completion of an intravenous glucose tolerance test, as reported elsewhere (17), participants were provided with a low-fat meal (2 MJ; <5 g fat) and a drink of water, and measures of vascular function a ≥2 h later were made at St Thomas’ Hospital between 1400 and 1700; both baseline and follow-up measures were made at the same time of day to minimize diurnal variations. Subjects were allowed to rest in the supine position in a temperature-controlled room (23°C) for 30 min before measurements of arterial stiffness and supine blood pressure were made. Arterial stiffness was estimated as PWV_c-f_ with the SphygmoCor Vx pulse-wave velocity system and software (SphygmoCor version 7.01; AtCor Medical Pty). PWV_c-f_ was computed from the time delay between the upstroke of the arterial pressure wave at the carotid and femoral arteries and the anatomical distance between the sternal notch and femoral pulse at the point of applanation. The CV for repeat measures on PWV_c-f_ was <3% on the same day and 7% in the same subject on different days. Measurements were made after 15 min supine rest with blood pressure recorded in triplicate by using an automated sphygmomanometer (Omron 705CP; Omron Healthcare); peripheral and central augmentation indexes were also determined with the SphygmoCor device by an analysis of the radial arterial waveform obtained by tonometry. Additional measures of arterial tone (20) were made by using photoplethysmography (PulseTrace; Micro Medical) and used to calculate the digital volume pulse reflection index and digital volume pulse stiffness index (DVP_SI_).

Endothelium and endothelium independent vasodilation was assessed by using a high-resolution ultrasound system with a 7–10-MHz linear array transducer (Acuson Aspen; Acuson Corp). Measurements were made by an experienced vascular ultrasonographer, who was blinded to treatment allocations, by using techniques previously described (21). FMD was expressed as the percentage increase in brachial artery diameter from baseline to maximal dilation that occurred 30–90 sec after the release of the cuff. The dilation to glycerol trinitrate was expressed as the percentage of increase in brachial artery diameter from baseline to maximal dilation after glycerol trinitrate. Reproducibility studies to assess the CV for repeated measures on the same subject indicated a CV ≤7% for measurements made on the same day and 18% for measurements made on different days.

### Statistical analyses

Data were analyzed by using ANCOVA by regressing the follow-up measure against baseline measure with ethnicity, BMI, and sex as covariates with SPSS/PC software (version 20.0; IBM Software). Box Cox regression models were used to select suitable data transformation and, when appropriate, analyses were attempted after a natural logarithmic transformation. A global test of between diet differences with 2 df that provide a *P <* 0.05 was prespecified as a condition to be met before additional between-diet differences were explored, and a Bonferroni correction was applied for multiple comparisons when appropriate. The effect of each diet was expressed as the change from the mean at baseline with 95% CIs. Linear regression modeling was undertaken by using the automatic linear modeling module in the SPSS software. Correlations between variables were made by using Pearson's product-moment correlation coefficient. The HOMA-IR was calculated as follows:
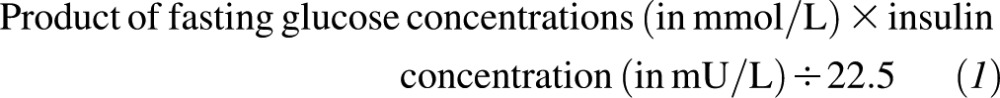


## RESULTS

**FIGURE 1.**
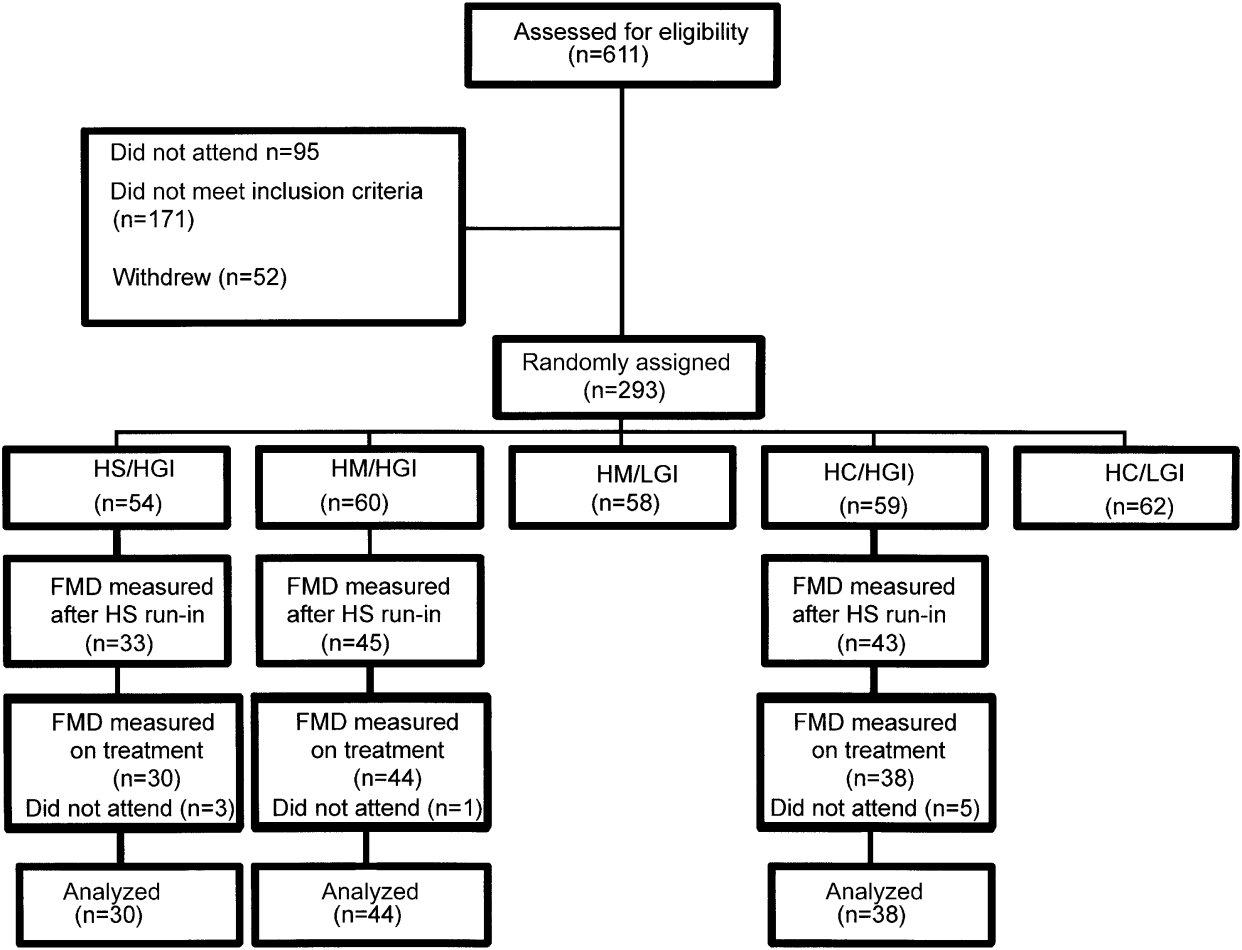
Consolidated Standards of Reporting Trials diagram showing flow of participants through the study. FMD was not measured in participants in the HM/LGI or HC/LGI groups. FMD, flow-mediated dilation; HC/HGI, high carbohydrate/high glycemic index; HC/LGI, high carbohydrate/low glycemic index; HM/HGI, high MUFA/high glycemic index; HM/LGI, high MUFA/low glycemic index; HS, high SFA; HS/HGI, high SFA/high glycemic index.

The flow of participants through the study is shown in **Figure 1**, and data were available for the analysis of the primary outcome in 112 participants, and their details by treatment allocation are shown in **Table 1**. There were more women than men, and most of the women were postmenopausal. Approximately one-fifth of the study population was nonwhite with similar proportions of Asian and black participants. Most participants were overweight or obese or had waist circumferences greater than the cutoffs used to indicate risk of metabolic syndrome (94 cm in men and 80 cm in women), and fasting insulin was elevated as was HOMA-IR, which indicated insulin resistance. Smoking prevalence, which was confirmed by cotinine measurements, was low. Dietary intakes after the run-in period and the mean of 2 measurements made during the intervention are shown in **Table 2**. Compared with the HS reference diet, SFA intakes fell to <10% of energy, and the intake of MUFAs was greater with the MUFA diet, and the intake of carbohydrates was greater with HC diets. The intake of PUFAs was lower with the HC than MUFA diet, but the size of the difference was small, and analyses of the plasma phospholipid composition showed no differences between treatments in proportions of PUFAs (*see* Supplementary Table 1 under “Supplemental data” in the online issue).

**TABLE 1:** Details of subjects after a 4-wk run-in period of an SFA-rich reference diet according to randomly assigned treatment with an HS, HM, or HC*^1^*

	HS (*n* = 30)	HM (*n* = 44)	HC (*n* = 38)
Age (y)	51 ± 7.9*^2^*	51 ± 10.2	51 ± 9.2
Sex [*n* (%)]			
M	12 (40)	14 (32)	14 (37)
F	18 (60)	30 (68)	24 (63)
Postmenopausal [*n* (%)]	11 (61)	11 (37)	14 (58)
Ethnicity [*n* (%)]			
White	17 (57)	25 (57)	26 (68)
Black	3 (10)	4 (9)	7 (18)
Asian	6 (20)	14 (31)	4 (17)
Far Eastern	1 (3)	1 (2)	0 (0.0)
Other	3 (10)	0 (0)	1 (3)
BMI (kg/m^2^)	28.5 ± 4.3	28.5 ± 4.5	27.7 ± 3.8
Waist circumference (cm)			
F	95 ± 12	96 ± 12	95 ± 11
M	101 ± 7.4	104 ± 6.8	101 ± 8.0
Systolic BP (mm Hg)	127 ± 16.1	123 ± 13.1	127 ± 17.7
Diastolic BP (mm Hg)	78 ± 10.3	79 ± 9.1	77 ± 9.6
Glucose (mmol/L)	5.6 ± 0.7	5.5 ± 0.6	5.3 ± 0.5
Insulin (mU/L)	9.1 ± 4.4	10.4 ± 8.1	7.5 ± 3.8
HOMA-IR	2.4 ± 1.5	2.3 ± 1.4	1.8 ± 1.1
ISI (×10^−4^ mL · μU^−1^ · min^−1^)	3.4 ± 2.4	3.0 ± 1.9	4.0 ± 2.1
Total cholesterol (mmol/L)	5.6 ± 0.9	5.3 ± 0.9	5.3 ± 0.9
Triacylglycerol (mmol/L)	1.5 ± 0.6	1.5 ± 0.6	1.1 ± 0.3
HDL cholesterol, women (mmol/L)	1.31 ± 0.22	1.29 ± 0.27	1.43 ± 0.28
HDL cholesterol, men (mmol/L)	1.25 ± 0.27	1.14 ± 0.23	1.24 ± 0.27
Smoking habit [*n* (%)]	5 (17)	2 (5)	2 (5)
BP medication [*n* (%)]	3 (10)	6 (14)	4 (11)

1 There were no significant differences by treatment allocation (chi-square and Mann-Whitney *U* tests). BP, blood pressure; HC, low-SFA/high-carbohydrate diet; HM, low-SFA/high-MUFA diet; HS, high-SFA diet; ISI, insulin sensitivity index from a short intravenous glucose tolerance test.

2 Mean ± SD (all such values).

**TABLE 2:** Macronutrient intakes and body weights in study participants at baseline after a 4-wk run-in period of an SFA-rich reference diet and at 24 wk after random assignment to an HS, HM, or HC*^1^*

	HS	HM	HC
Energy (MJ/d)			
HS run-in	7.74 ± 2.27	7.61 ± 2.21	7.70 ± 2.24
Follow-up	7.90 ± 2.41	7.99 ± 2.18	8.13 ± 2.21
Protein (% of energy)			
HS run-in	17.1 ± 3.8	16.3 ± 3.0	16.4 ± 3.1
Follow-up	17.8 ± 3.8	16.2 ± 3.6	17.8 ± 3.3
Carbohydrate (% of energy)			
HS run-in	44.6 ± 7.3	45.9 ± 8.0	44.4 ± 5.9
Follow-up	42.8 ± 7.3	47.2 ± 7.4	53.1 ± 6.3*^2^*
Fat (% of energy)			
HS run-in	35.7 ± 5.3	35.5 ± 6.1	36.9 ± 3.5
Follow-up	35.7 ± 5.3	34.4 ± 7.4	26.1 ± 5.4*^2^*
SFA (% of energy)			
HS run-in	15.3 ± 3.0	14.5 ± 3.0	15.1 ± 2.4
Follow-up	14.6 ± 2.8	9.4 ± 2.7*^3^*	8.5 ± 2.0*^3^*
MUFA (% of energy)			
HS run-in	11.3 ± 2.4	10.8 ± 2.4	11.7 ± 1.5
Follow-up	11.3 ± 2.5	15.0 ± 4.5*^4^*	9.3 ± 2.6
PUFA (% of energy)			
HS run-in	5.6 ± 1.1	6.3 ± 2.1	6.1 ± 1.1
Follow-up	5.7 ± 1.4	6.5 ± 1.9	5.3 ± 1.9*^5^*
Body weight (kg)			
HS run-in	77.9 ± 13.9	78.3 ± 15.2	78.5 ± 14.0
Follow-up	78.1 ± 14.1	77.2 ± 15.7	77.9 ± 14.4

1 All values are means ± SDs. Data were analyzed by using ANCOVA with the run-in value as a covariate. In HS, HM, and HC groups, *n* = 28, *n* = 41, and *n* = 35, respectively, for dietary intake, except for weight, for which *n* = 30, *n* = 44, and *n* = 38, respectively. HC, low-SFA/high-carbohydrate diet; HM, low-SFA/high-MUFA diet; HS, high-SFA diet.

2 *^2^P* < 0.01 compared with HS and HM by using Bonferroni's multiple-comparison test.

3 *^3^P* < 0.01 compared with HS by using Bonferroni's multiple-comparison test.

4 *^4^P* < 0.01 compared with HS and HC by using Bonferroni's multiple-comparison test.

5 *^5^P* < 0.05 compared with HM by using Bonferroni's multiple-comparison test.

Results for primary and secondary outcomes are shown in **Table 3**, and results for exploratory outcomes are shown **Table 4**. The mean value for FMD was similar to that reported by us in previous studies in subjects of the same age from our group (19, 21), with mean values 0.7% lower in men [6.2% (95% CI: 5.7, 6.0) in men compared with 6.9% (95% CI: 6.5, 7.4) in women]. Only 11.6% of participants had impaired FMD <4%, which, in our laboratory, is considered to indicate impaired endothelial function, at baseline and 12.5% at follow-up. Regression analysis showed that 23% of the variation in FMD could be explained as the percentage of this variance by differences in age (24%), systolic blood pressure (48%), metabolic score (18%), and BMI (12%). No relation was shown with fasting insulin or measures of insulin sensitivity and FMD. There were no changes in FMD or endothelium independent dilation with the dietary intervention. At baseline, the geometric mean (±SD) for PWV_c-f_ was 7.67 ± 1.62 m/s, and 50.3% of the variance could be explained as percentages of this variance by differences of systolic blood pressure (48%), age (35%), and BMI (16%). PWV_c-f_ was negatively correlated with FMD (*r* = 0.318; *P* < 0.001) and positively correlated with DVP_SI_ (*r* = 0.434, *P* < 0.001). However, the latter was not correlated with FMD. There were no significant differences between treatments on PWV_c-f_, supine blood pressure, plasma 8-isoprostane F2α-III concentration, central and peripheral augmentation indexes, digital volume pulse reflection index, or DVP_SI_. However, there was a trend for DVP_SI_ to be lower with the HC than HS diets.

**TABLE 3:** Endothelium-dependent and -independent vasodilation, blood pressure, arterial stiffness, and plasma 8-isoprostane F_2α_-III concentrations at baseline after a 4-wk run-in period of an SFA-rich reference diet and at 24 wk after random assignment to an HS, HM, or HC*^1^*

	HS (*n* = 30)	HM (*n* = 44)	HC (*n* = 38)	*P*
Baseline arterial diameter (mm)				
HS run-in	3.62 ± 0.61*^2^*	3.64 ± 0.60	3.65 ± 0.79	—
Follow-up	3.75 ± 0.99	3.60 ± 0.56	3.58 ± 0.72	—
Change with treatment*^3^*	0.13 (−03, 0.29)	−0.03 (−0.17, 0.10)	−0.08 (−0.22, 0.10)	0.164
Flow-mediated dilation (endothelium dependent) (%)				
HS run-in	6.4 ± 2.3	6.9 ± 2.3	6.6 ± 2.2	—
Follow-up	6.8 ± 2.3	6.8 ± 2.4	6.7 ± 2.4	—
Change with treatment*^3^*	0.3 (−0.4, 1.1)	−0.2 (−0.8, 0.5)	0.1 (−0.6, 0.7)	0.79
GTN-mediated dilation (endothelium independent) (%)				
HS run-in	11.2 ± 3.1	11.4 ± 3.1	11.5 ± 4.4	—
Follow-up	11.1 ± 3.6	11.7 ± 3.3	11.2 ± 3.8	—
Change with treatment*^3^*	−0.1 (−1.3, 1.2)	0.3 (−0.8, 1.4)	−0.3 (−1.4, 0.9)	0.56
PWV_c-f_ (arterial stiffness) (m/s)*^4^*				
HS run-in	8.20 (7.61, 8.83)	7.51 (7.07, 7.98)	7.47 (6.99, 7.97)	—
Follow-up	8.13 (7.55, 8.73)	7.72 (7.27, 8.19)	7.40 (6.94,7.89)	—
Percentage of change with treatment	−1.0 (−6.2, 4.3)	2.7 (−1.4, 6.9)	−1.0 (−5.5, 3.4)	0.62
Supine systolic BP (mm Hg)				
HS run-in	119.0 ± 18.2	115.3 ± 12.5	116.7 ± 15.3	—
Follow-up	118.4 ± 14.7	115.5 ± 12.4	113.1 ± 12.3	—
Change with treatment*^3^*	−0.5 (−4.2, 3.2)	0.1 (−2.9, 3.2)	−3.6 (−6.9, −0.4)	0.07
Supine diastolic BP (mm Hg)				
HS run-in	72.2 ± 8.9	71.8 ± 6.1	70.5 ± 8.9	—
Follow-up	70.8 ± 8.8	69.2 ± 6.5	67.7 ± 7.1	—
Change with treatment*^3^*	−1.4 (−3.4, 0.5)	−2.7 (−4.2, −0.5)	−2.8 (−4.6, −1.1)	0.30
Plasma 8-isoprostane F_2α_-III (pmol/L)*^4^*				
HS run-in	175 (161, 213)	175 (155, 204)	180 (160, 203)	—
Follow-up	176 (155, 198)	185 (166, 205)	188 (168, 210)	—
Percentage of change with treatment	1 (−12, 14)	6 (−5, 16)	4 (−7, 16)	0.71

1 *P* values are for treatment effects expected in the 3 groups derived from ANCOVA of the value with treatment regressed against age, sex, BMI, and ethnicity and the value at run-in. BP, blood pressure; GTN, glycerol trinitrate; HC, low-SFA/high-carbohydrate diet; HM, low-SFA/high-MUFA diet; HS, high-SFA diet; PWV_c-f_, carotid to femoral pulse wave velocity.

2 Mean ± SD (all such values).

3 All values are means; 95% CIs in parentheses.

4 All values are geometric means; 95% CIs in parentheses. Data were log transformed.

**TABLE 4:** Changes in central and peripheral augmentation indexes, digital volume pulse stiffness, and reflection indexes at baseline after a 4-wk run-in period of an SFA-rich reference diet and at 24 wk after random assignment to an HS, HM, or HC*^1^*

	HS (*n* = 30)	HM (*n* = 44)	HC (*n* = 38)	*P*
Central AIX (%)				
HS run-in	28.6 ± 14.2*^2^*	26.1 ± 9.6	22.1 ± 11.2	—
Follow-up	29.4 ± 21.4	25.2 ± 15.4	20.3 ± 10.8	—
Change with treatment*^3^*	0.85 (−4.1, 5.8)	−0.9 (−4.1, 5.8)	−1.8 (−4.1, 5.8)	0.23
Peripheral AIX (%)				
HS run-in	77.8 ± 20.73	76.8 ± 14.69	72.5 ± 15.5	—
Follow-up	72.3 ± 17.74	73.4 ± 16.94	68.7 ± 14.5	—
Percentage change*^4^*	−5.59 (−11.2, 0.1)	−3.42 (−8.0, 1.2)	−4.12 (−8.8, 1.2)	0.96
DVP_SI_ (m/s)				
HS run-in	7.89 ± 1.56	8.15 ± 1.97	8.11 ± 2.29	—
Follow-up	8.46 ± 1.96	8.07 ± 2.06	7.54 ± 1.92	—
Change with treatment*^3^*	0.57 (−0.15, 1.29)	−0.08 (−0.67, 0.50)	−0.57 (−1.20, 0.06)	0.055
DVP_RI_ (%)				
HS run-in	77.24 ± 9.04	75.05 ± 8.69	71.11 ± 9.86	—
Follow-up	74.42 ± 9.49	72.78 ± 8.61	68.71 ± 12.73	—
Change with treatment*^3^*	−2.82 (6.46, 0.82)	−2.27 (−5.20, 0.67)	−2.40 (−5.64, 0.83)	0.30

1 *^1^P* values are for treatment effects expected in the 3 groups derived from ANCOVA of the value with treatment regressed against age, sex, BMI, and ethnicity and the value at run-in. AIX, augmentation index; DVP_RI_, digital volume pulse reflection index; DVP_SI_, digital volume pulse stiffness index; HC, low-SFA/high-carbohydrate diet; HM, low-SFA/high-MUFA diet; HS, high-SFA diet.

2 Mean ± SD (all such values).

3 All values are means; 95% CIs in parentheses.

4 All values are geometric means; 95% CIs in parentheses. Data were log transformed.

## DISCUSSION

In this study, we recruited subjects who were moderately insulin resistant but not diabetic. We expected to find a significant proportion of participants with impaired FMD, but surprisingly, only 11–12% of subjects had FMD values <4%. This result was in contrast to our finding in the Modulation of Atherosclerotic Risk by Increasing dose of N-3 fatty Acids study (21), in which we showed the proportion with impaired FMD to be 45% of men and 33% of women, who were of a similar age but less insulin resistant (HOMA-IR: 1.4 compared with 2.2 in the current study). A recent cross-sectional study of 3516 participants in the United States (22) showed the mean (±SD) FMD to be 4.8 ± 3.0% in white Americans but higher in subjects with impaired glucose fasting glucose (5.6–6.9 mmol/L), which were consistent with our findings. However, in the study (22), clear evidence was shown that FMD is impaired in patients with type 2 diabetes whose fasting glucose concentration was >6.9 mmol/L. In the current study, we set out to test the hypothesis that decreasing SFA intake would improve vascular function. Our hypothesis was based on the belief that decreasing SFA intake would improve insulin sensitivity, which the main report (17) showed not to be the case.

Strengths of the current study were its relatively large size and duration of 24 wk. Furthermore, all participants were run in for 4 wk after receiving an HS diet before being allocated to the various treatments, which eliminated any bias attributable to a variation in the background diet. We were careful to use a standardized protocol that used an experienced ultrasonographer who was blinded to the treatment allocation. The study was powered to detect a 1% FMD unit change between diets. Mean observed changes from baseline differed by ≤0.3% FMD units on follow-up, well below the 1% change in FMD that was chosen to represent the smallest clinically relevant change. It is unlikely that an increase in sample size would have revealed different results. The study by Keogh et al (11) conducted in Australia had a crossover design, and participants were of similar age, BMI, and lipid profile as in the current study. The dietary intervention involved exchanging 20 g high-PUFA margarine and 35 g walnuts (PUFA) or 20 g HM margarine and 45 g almonds (MUFA) or 50 g butter (SFA) or 70 g sultanas and 50 g jam (HC) in the diet of participants for 3-wk periods. The mean FMD was 5.41 ± 2.45% with the SFA diet compared with 10.80 ± 3.69% with the other diets, and there were no differences between the HC diet and other MUFA or PUFA diets. However, the interpretation of the results of that study were confounded by the use of high intakes of walnuts, ,almonds, and sultanas, which contain polyphenolic compounds that have antioxidant and other pharmacologic properties and can influence endothelial function (23).

In this context, it is relevant to consider the beneficial effects on FMD that have been reported in subjects given virgin olive oil (12, 14, 15, 24), which are now being attributed to its polyphenol content rather than its HM content. We were careful to standardize the vitamin E content of fats used in our study and used the same ratio of linoleic:linolenic acid in fat spreads. Our dietary model was based on exchanges of everyday foods and the manipulation of their fat contents and compositions. The lack of any change in FMD in the current study was also consistent with the lack of any change in 8-isoprostane F2α-III concentrations, which indicated no difference in the production of reactive oxygen species between the 3 treatments. Arterial stiffness as measured by PWV_c-f_ is regarded as an index of arterial ageing, is easier to measure than FMD, and may be a better predictor for future cardiovascular disease (2). In this study, although we were unable to show any effect of dietary fat intake on PWV_c-f_, it needs to be acknowledged that arterial stiffening occurs slowly, and a much-longer period of intervention may be necessary to show changes.

The replacement of SFAs with either MUFAs or carbohydrates has been widely advocated for the prevention of cardiovascular disease because 12–16-carbon SFAs elevate serum total- and LDL-cholesterol concentrations, although the effects on the ratio of total cholesterol:HDL cholesterol, which is regarded to be a more- robust indicator (25) of risk, is modest (26). However, a meta-analysis of data from cohort studies (27) have not shown an association between SFA intake and cardiovascular disease on the basis of clinical endpoints. Furthermore, although a meta-analysis of prospective cohort studies failed to show a benefit in terms of reduced risk of mortality from coronary heart disease when SFAs were replaced with PUFAs, this was not the case when SFAs were replaced with MUFAs or carbohydrates (28). In the current study, we were unable to show any benefit on vascular function from replacing SFAs with MUFAs or carbohydrates. In the current study much of the saturated fat in the diet was provided by palm oil and milk fat rather than meat fats, and the MUFA was provided by refined high oleic sunflower oil. However, it may well be that sources of MUFA or PUFA used to replace animal fats in the diet may contain non–fatty acid components that influence endothelial function and, in the future, closer attention needs to be focused on these components, especially in view of a recent report that showed beneficial effects of a Mediterranean diet rich in virgin olive oil on stroke (29).

## Supplementary Material

Supplemental data

## Footnotes

4 Abbreviations used: DVP_SI_, digital volume pulse stiffness index; FMD, flow-mediated dilation; HC, high carbohydrate; HM, high MUFA; HS, high SFA; NO, nitric oxide; PWV_c-f_, carotid to femoral pulse wave velocity.
