# Supplementary material for: SFAs do not impair endothelial function and arterial stiffness1
Source: Am J Clin Nutr. 2013 Aug 14;98(3):677–83. doi: 10.3945/ajcn.113.063644 (PMC3743730; doi:10.3945/ajcn.113.063644)
Supplement: Supplemental data [file supp_98_3_677__index.html]

Supplemental data 

# SFAs do not impair endothelial function and arterial stiffness

## Supplemental data

**Files in this Data Supplement:**

- Supplemental data - Text 1
- Supplemental data - Table 1
