# Supplementary material for: SFAs do not impair endothelial function and arterial stiffness1
Source: Am J Clin Nutr. 2013 Aug 14;98(3):677–83. doi: 10.3945/ajcn.113.063644 (PMC3743730; doi:10.3945/ajcn.113.063644)
Supplement: Supplemental data [file 98.3.677_ajcn063644SupplementaryData1.pdf]

Dietary advice given in RISCK study to participants on the high glycemic index arms

Diet A= high saturated fat reference diet (HM)

Diet B= high monounsaturated fat diet (HM)

Diet D=high carbohydrate diet (HC)

SOME OF YOUR QUESTIONS ANSWERED

RISCK
READING/IMPERIAL/SURREY/CAMBRIDGE/KINGS

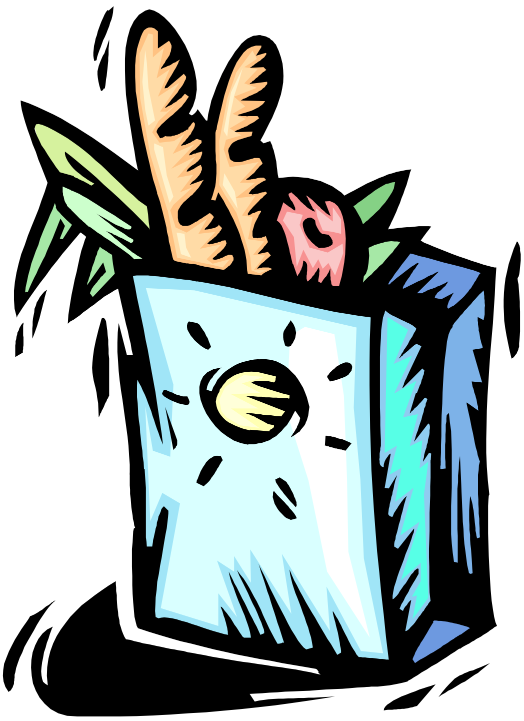

YOUR DIETARY ADVICE

Thank you for volunteering to take part in the RISCK study. Please take the time to read this information booklet, which gives you advice on the changes we will be asking you to make to your diet during the study.

Why are we asking you to make changes to your diet?

The aim of this study is to find the best dietary approach to reduce the risk for developing diseases such as diabetes and heart disease in the future. **This is not a weight loss study** and you are free to eat as much or as little as you wish of other foods. We will examine whether specific changes in the amounts and types of food you eat can improve your health.

Why is it important that I make the changes to my diet?

It is now widely recognised that there are links between what we eat and our health. This research project will provide important guidance on the types and quantities of foods we should be eating to improve health. We recognise that the success of the study is wholly dependent on people, like you, volunteering their time to take part. However the results we obtain will only be useful to public health recommendations if we can be sure that the dietary changes we ask you to make have been followed.

What support will I receive to help me follow this dietary advice?

We will help you to follow your dietary advice by providing a number of foods we would like you to introduce into your diet. If you are unable to eat the foods we provide it is important to tell us and we will try our best to offer alternatives so that you may be able to continue to take part.

What changes will I have to make to my diet?

The study will be comparing five different diets with varying types and amounts of fat and carbohydrate. You have been allocated to a group that will follow one of these diets; this was a random process meaning that you had an equal chance of being allocated to any one of the five groups. These guidelines are specific to the group to which you have been allocated. Each of the five groups is equally important to the findings of the study.

During the next 7 months we need you to follow your normal diet but will ask you to replace a number of specific foods and to avoid others.

We will be asking you to eat certain foods in particular quantities, these are everyday foods and do not contain any unusual ingredients and will replace similar foods that you are likely to eat as part of your normal diet. In addition we will be asking you to avoid certain foods that you may or may not regularly eat. These changes to your diet will ensure that the types and amounts of fat and carbohydrate in your diet match the requirements of the group to which you have been allocated. During the study we will ask you to make changes to your consumption of the following foods, the majority of which we will provide for you.

1. Fat spreads
2. Cooking oils
3. Dairy foods
4. Breakfast cereals
5. Bread
6. Rice/pasta/potatoes
7. Snacks

HOW TO FOLLOW THE DIETARY GUIDELINES

In this booklet we have firstly included advice on the collection and provision of each of the specific foods we will be asking you eat during the study. This is contained in the section '**Collection and provision of foods**.'

We have also provided advice for each food group on:

1. How much of each of these foods you should eat each day
2. The foods you should choose whilst taking part in the study
3. The foods you should avoid whilst taking part in the study

We can provide laminated copies of this information if you would like which you can keep in your kitchen, perhaps stick to your fridge, to remind you of what foods you should and shouldn't be eating and in what quantities.

Collection and provision of foods

We will ask you to collect foods from the study centre every two weeks. We will try and make alternative arrangements if these collection times are difficult for you.

We will provide some of the study foods for your use only. Other foods, generally used in cooking and preparation of meals, will be provided in sufficient quantities for your whole household.

Just For You

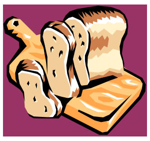

Bread. Your study centre will provide this. As bread has a limited shelf life this will be provided frozen and may be stored in your freezer until you are ready to eat.

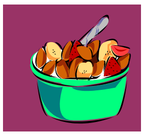

Breakfast cereals Your study centre will provide these.

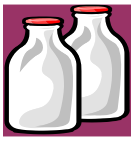

Dairy foods. We will ask you to purchase these foods but have included details and examples of the types that we would like you to choose in this booklet.

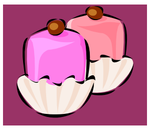

Snacks: Your study centre will provide these.

For your Household

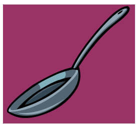

Fat Spreads and cooking Oils. Your study centre will provide these. They may be used in the preparation of meals and baking.

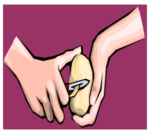

Rice and potatoes. Your study centre will provide these.

Daily Intakes

<table>
<tr><th>Study Foods</th><th colspan="2">Quantities</th><th>Advice on how to achieve</th></tr>
<tr><td rowspan="2">Fat Spreads</td><td>Men</td><td>4 teaspoons</td><td rowspan="2">Use fat spreads on each slice of bread you eat. If you do not use your daily amount of spread on bread, add the rest to other foods such as jacket potatoes and vegetables and in cooking and baking.*</td></tr>
<tr><td>Women</td><td>3 teaspoons</td></tr>
<tr><td rowspan="2">Cooking Fats</td><td>Men</td><td>1 tblespoon</td><td rowspan="2">You should aim to use the cooking fats in cooking and baking.*</td></tr>
<tr><td>Women</td><td>2 teaspoons</td></tr>
<tr><td>Salad Dressing</td><td colspan="3">A simple Crème Fraiche dressing can be made with this recipe:
1/4 cup white-wine vinegar
1 tablespoon sugar
1 1/2 cups creme fraiche or sour cream
Freshly ground pepper
Plus other seasonings of your choice</td></tr>
<tr><td rowspan="2">Cheese</td><td>Men</td><td colspan="2">We do not need you to eat cheese everyday but aim to include three cheese sandwiches a week (with a generous portion of cheese). Alternatively use a similar quantity of cheese in recipes.*</td></tr>
<tr><td>Women</td><td colspan="2">We do not need you to eat cheese everyday but aim to include two cheese sandwiches a week (with a generous portion of cheese). Alternatively use a similar quantity of cheese in recipes.*</td></tr>
<tr><td>Milk</td><td>Men & Women</td><td colspan="2">Please use milk on the cereal provided, in your tea and coffee and in any recipes including milk.* If you do not drink tea or coffee, aim to have a small-medium glass of milk/day.</td></tr>
<tr><td>Breakfast cereals/ Bread/Rice/Potatoes</td><td>Men & Women</td><td colspan="2">Please eat the study breakfast cereals, bread, rice and potatoes in the same quantities you eat in your usual diet less 1 portion/day of <u>one</u> of the foods, see below.
Carbohydrate Portions:
<table>
<tr><th rowspan="2">Food</th><th colspan="2">1 portion =</th><th rowspan="2">Measures:</th></tr>
<tr><th>Men</th><th>Women</th></tr>
<tr><td>Breakfast cereals</td><td>6</td><td>4</td><td>heaped tablespoons</td></tr>
<tr><td>Study Bread</td><td>1½</td><td>1</td><td>medium slice</td></tr>
<tr><td>French Bread</td><td>2</td><td>1½</td><td>Inch slice</td></tr>
<tr><td>Study Rice, cooked</td><td>3</td><td>2</td><td>heaped tablespoons</td></tr>
<tr><td>Potatoes</td><td>1½</td><td>1</td><td>egg-sized potato</td></tr>
<tr><td>Chappati, wheat flour</td><td>1½</td><td>1</td><td>Small (6” diameter</td></tr>
</table>
Avoid pasta.</td></tr>
<tr><td>Snacks</td><td>Men & Women</td><td colspan="2">Please replace one snack that you normally eat during the day with one of the biscuits we provide</td></tr>
</table>

- * Please remember if you use the study fat spreads, oils, cheese and milk to make meals to be eaten not only by yourself but other members of your family/household, you will need to divide the amounts added to the recipe by the number of people consuming the meal to calculate how much **you yourself have eaten**.
- * For example if you use a tablespoon of oil in a recipe for two people including yourself you will have eaten about half a tablespoon.

Foods to Choose/Avoid

Eating at home

	Choose	Avoid
**Spreads**	Spread provided	All other types of spread
**Cooking oils**	Oils provided	All other types of cooking oil
**Cheese**	Full fat	Half-fat or reduced fat including Heinz half fat cheddar, Tesco healthy eating/living varieties, Sainsbury's be good to yourself varieties, Asda 'Good for you' varieties and cottage cheese.
**Milk**	Whole<br>Yoghurt	Skimmed, semi skimmed or soya
**Breakfast cereals**	Cereals provided (Branflakes Rice Crispies, Balance)	All other breakfast cereals
**Bread**	Bread provided or French bread. Other types of bread in smaller quantities. Wholemeal	
**Rice/Pasta/Potatoes**	Rice provided and potatoes	Basmati rice and all types of pasta
**Snacks**	Biscuits provided (to replace one usually eaten snack/day).	

Eating out

	Foods to choose	Foods to avoid
**Spreads**		Avoid all spreads
**Cooking oils**		Avoid adding additional oils or salad dressings.
**Cheese**	Full fat varieties	Avoid cheese if type unspecified and low fat varieties including cottage cheese
**Milk**	Ask for whole milk	Skimmed, semi-skimmed or soya
**Breakfast cereals**	Choose Branflakes or Rice Crispies.	Avoid all other breakfast cereals
**Bread**	Any types of bread	
**Rice/Pasta/Potatoes**	Choose potatoes or bread as an accompaniment.	Avoid dishes with pasta or basmati rice (choose naan bread rather than rice in Indian restaurants). Noodles

SOME OF YOUR QUESTIONS ANSWERED

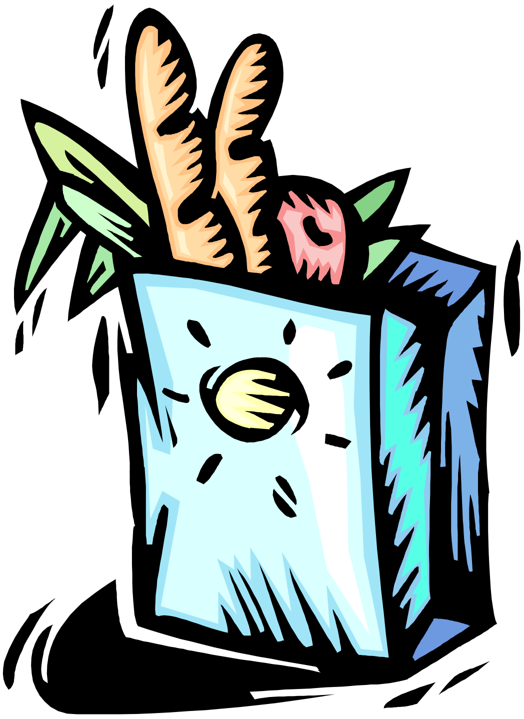

YOUR DIETARY ADVICE

Thank you for volunteering to take part in the RISCK study. Please take the time to read this information booklet, which gives you advice on the changes we will be asking you to make to your diet during the study.

B

Why are we asking you to make changes to your diet?

The aim of this study is to find the best dietary approach to reduce the risk for developing diseases such as diabetes and heart disease in the future. **This is not a weight loss study** and you are free to eat as much or as little as you wish of other foods. We will examine whether specific changes in the amounts and types of food you eat can improve your health.

Why is it important that I make the changes to my diet?

It is now widely recognised that there are links between what we eat and our health. This research project will provide important guidance on the types and quantities of foods we should be eating to improve health. We recognise that the success of the study is wholly dependent on people, like you, volunteering their time to take part. However the results we obtain will only be useful to public health recommendations if we can be sure that the dietary changes we ask you to make have been followed.

What support will I receive to help me follow this dietary advice?

We will help you to follow your dietary advice by providing a number of foods we would like you to introduce into your diet. If you are unable to eat the foods we provide it is important to tell us and we will try our best to offer alternatives so that you may be able to continue to take part.

What changes will I have to make to my diet?

The study will be comparing five different diets with varying types and amounts of fat and carbohydrate. You have been allocated to a group that will follow one of these diets; this was a random process meaning that you had an equal chance of being allocated to any one of the five groups. These guidelines are specific to the group to which you have been allocated. Each of the five groups is equally important to the findings of the study.

During the next 6 months we need you to follow your normal diet but will ask you to replace a number of specific foods and to avoid others.

We will be asking you to eat certain foods in particular quantities, these are everyday foods and do not contain any unusual ingredients and will replace similar foods that you are likely to eat as part of your normal diet. In addition we will be asking you to avoid certain foods that you may or may not regularly eat. These changes to your diet will ensure that the types and amounts of fat and carbohydrate in your diet match the requirements of the group to which you have been allocated. During the study we will ask you to make changes to your consumption of the following foods, the majority of which we will provide for you.

1. Fat spreads
2. Cooking oils
3. Dairy foods
4. Breakfast cereals

5. Bread
6. Rice/pasta/potatoes
7. Salad dressings
8. Snacks

In this booklet we have firstly included advice on the collection and provision of each of the specific foods we will be asking you eat during the study. This is contained in the section '**Collection and provision of foods**.'

HOW TO FOLLOW THE DIETARY GUIDELINES

These are your diet guidelines for Months 2 – 7 of the study

In these sections we have provided advice for each food group on:

1. How much of each of these foods you should eat each day
2. The foods you should choose whilst taking part in the study
3. The foods you should avoid whilst taking part in the study

Collection and provision of foods

We will ask you to collect foods from the study centre every two weeks. We will try and make alternative arrangements if these collection times are difficult for you.

We will provide some of the study foods for only you to use (i.e. Bread, Breakfast cereal, and Snacks).

Other foods, generally used in cooking and preparation of meals, will be provided in sufficient quantities for your whole household (i.e. Fat spreads and cooking oils and rice and potatoes).

Daily Intakes

<table>
<tr><th>Study Foods</th><th colspan="2">Quantities</th><th>Advice on how to achieve</th></tr>
<tr><td rowspan="2">Fat Spreads</td><td>Men</td><td>4 teaspoons</td><td rowspan="2">Use fat spreads on each slice of bread you eat. If you do not use your daily amount of spread on bread, add the rest to other foods such as jacket potatoes, vegetables and in cooking and baking.*</td></tr>
<tr><td>Women</td><td>3 teaspoons</td></tr>
<tr><td rowspan="2">Cooking Oils</td><td>Men</td><td>1 tablespoon</td><td rowspan="2">You should aim to use the oils in cooking or to make a salad dressing*</td></tr>
<tr><td>Women</td><td>2 teaspoons</td></tr>
<tr><td>Cheese</td><td>Men & Women</td><td colspan="2">We do not need you to eat specific amounts of cheese but when you do eat cheese please select low fat varieties as shown in the Foods to Choose/Avoid table.</td></tr>
<tr><td>Milk</td><td>Men & Women</td><td colspan="2">Please use milk on the cereal provided, in your tea and coffee and in any recipes including milk.*</td></tr>
<tr><td>Breakfast cereals/ Bread/Rice/Potatoes</td><td>Men & Women</td><td colspan="2">Please eat study breakfast cereals, bread, rice and potatoes in the same quantities you eat in your usual diet less 1 portion/day of <u>one</u> of the foods, see below.
i.e. Please eat _______carbohydrate portions per day
<table>
<tr><th rowspan="2">Food</th><th colspan="2"></th><th rowspan="2">Measures:</th></tr>
<tr><th>Men</th><th>Women</th></tr>
<tr><td>Breakfast cereals</td><td>6</td><td>4</td><td>heaped tablespoons</td></tr>
<tr><td>Study Bread</td><td>1½</td><td>1</td><td>medium slice</td></tr>
<tr><td>French Bread</td><td>2</td><td>1½</td><td>inch slice</td></tr>
<tr><td>Study Rice, cooked</td><td>3</td><td>2</td><td>heaped tablespoons</td></tr>
<tr><td>Potatoes</td><td>1½</td><td>1</td><td>egg-sized potato</td></tr>
<tr><td>Chapatti, wheat flour</td><td>1½</td><td>1</td><td>small (6” diameter)</td></tr>
</table>
Avoid pasta.</td></tr>
<tr><td>Snacks</td><td>Men & Women</td><td colspan="2">Please replace one snack that you normally eat during the day with one of the biscuits we provide</td></tr>
<tr><td>Additional foods</td><td>Men & Women</td><td colspan="2">Every day we would also like you to eat 1 tablespoon of the salad dressing provided OR 2 tablespoons of hazelnuts.</td></tr>
</table>

- * Please remember if you use the study fat spreads, oils, cheese and milk to make meals to be eaten not only by yourself but other members of your family/household, you will need to divide the amounts added to the recipe by the number of people consuming the meal to calculate how much **you yourself have eaten**.
- * For example if you use a tablespoon of oil in a recipe for two people including yourself you will have eaten about half a tablespoon.

Foods to Choose/Avoid

Eating at home

	Choose	Avoid
**Spreads**	Spread provided	All other types of spread
**Cooking oils**	Oils provided	All other types of cooking oil.
**Cheese**	Half-fat or reduced fat including Heinz half fat cheddar, Tesco healthy eating/living varieties, Sainsbury's be good to yourself, Asda 'Good for you' varieties, Edam & cottage cheese.	Full fat
**Milk**	Skimmed<br>Low fat yoghurt	Semi skimmed, whole and soya<br>Cream
**Breakfast cereals**	Cereals provided (Branflakes, Rice Crispies, Balance)	All other breakfast cereals
**Bread**	Bread provided or French bread. Other types of bread in smaller quantities.	
**Rice/Pasta/Potatoes**	Rice provided and potatoes	Basmati rice and all types of pasta
**Salad dressing**	Salad dressing provided	Any other types
**Snacks**	Biscuits provided or nuts (to replace one usually eaten snack/day).	

Eating out

	Foods to choose	Foods to avoid
**Spreads**		Avoid all spreads
**Cooking oils**		Avoid adding additional oils or salad dressings
**Cheese**	Low fat varieties if specified including cottage cheese.	Avoid cheese or cheese-based dishes/sauces if type is unspecified
**Milk**	Ask for skimmed milk	Whole milk, cream, cream-based desserts & sauces
**Breakfast cereals**	Choose Branflakes or Rice Crispies	Avoid all other types
**Bread**	Any types of bread	
**Rice/Pasta/Potatoes**	Choose potatoes or bread as an accompaniment.	Avoid dishes with pasta or basmati rice (choose naan bread rather than rice in Indian restaurants)
**Snacks**	Nuts, Fruit, Seeds<br>Walkers Lites crisps	

SOME OF YOUR QUESTIONS ANSWERED

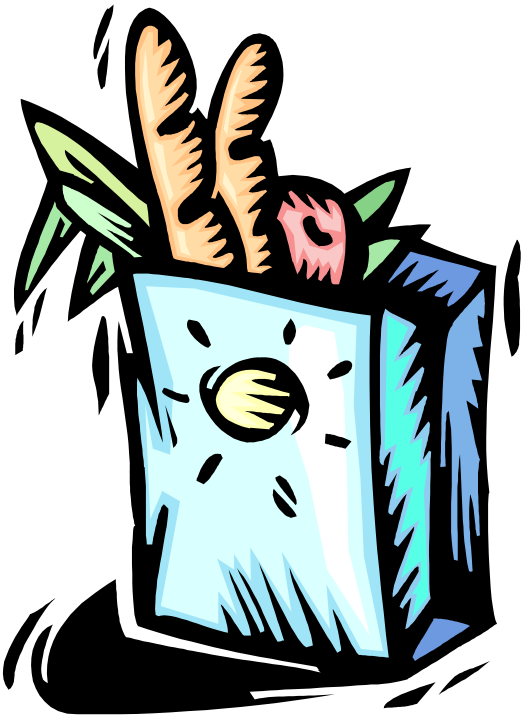

YOUR DIETARY ADVICE

Thank you for volunteering to take part in the RISCK study. Please take the time to read this information booklet, which gives you advice on the changes we will be asking you to make to your diet during the study.

Why are we asking you to make changes to your diet?

The aim of this study is to find the best dietary approach to reduce the risk for developing diseases such as diabetes and heart disease in the future. **This is not a weight loss study** and you are free to eat as much or as little as you wish of other foods. We will examine whether specific changes in the amounts and types of food you eat can improve your health.

Why is it important that I make the changes to my diet?

It is now widely recognised that there are links between what we eat and our health. This research project will provide important guidance on the types and quantities of foods we should be eating to improve health. We recognise that the success of the study is wholly dependent on people, like you, volunteering their time to take part. However, the results we obtain will only be useful to public health recommendations if we can be sure that the dietary changes we ask you to make have been followed.

What support will I receive to help me follow this dietary advice?

We will help you to follow your dietary advice by providing a number of foods we would like you to introduce into your diet. If you are unable to eat the foods we provide it is important to tell us and we will try our best to offer alternatives so that you may be able to continue to take part.

What changes will I have to make to my diet?

The study will be comparing five different diets with varying types and amounts of fat and carbohydrate. You have been allocated to a group that will follow one of these diets; this was a random process meaning that you had an equal chance of being allocated to any one of the five groups. These guidelines are specific to the group to which you have been allocated. Each of the five groups is equally important to the findings of the study.

During the next 6 months we need you to follow your normal diet but will ask you to replace a number of specific foods and to avoid others.

We will be asking you to eat certain foods in particular quantities, these are everyday foods and do not contain any unusual ingredients and will replace similar foods that you are likely to eat as part of your normal diet. In addition we will be asking you to avoid certain foods that you may or may not regularly eat. These changes to your diet will ensure that the types and amounts of fat and carbohydrate in your diet match the requirements of the group to which you have been allocated. During the study we will ask you to make changes to your consumption of the following foods, the majority of which we will provide for you.

1. Fat spreads
2. Cooking oils
3. Dairy foods
4. Breakfast cereals
5. Bread
6. Rice/pasta/potatoes

7. Snacks

In this booklet we have firstly included advice on the collection and provision of each of the specific foods we will be asking you eat during the study. This is contained in the section '**Collection and provision of foods**.'

HOW TO FOLLOW THE DIETARY GUIDELINES

These are your diet guidelines for Months 2 – 7 of the study

1. How much of each of these foods you should eat each day
2. The foods you should choose whilst taking part in the study
3. The foods you should avoid whilst taking part in the study

Collection and provision of foods

We will ask you to collect foods from the study centre every two weeks. We will try and make alternative arrangements if these collection times are difficult for you.

We will provide some of the study foods for only you to use (i.e. Bread, Breakfast cereal, and Snacks).

Other foods, generally used in cooking and preparation of meals, will be provided in sufficient quantities for your whole household (i.e. Fat spreads and cooking oils and rice and potatoes).

Daily Intakes

<table>
<tr><th>Study Foods</th><th colspan="2">Quantities</th><th>Advice on how to achieve</th></tr>
<tr><td rowspan="2">Fat Spreads</td><td>Men</td><td>No more than 4 teaspoons</td><td rowspan="2">Use fat spreads on bread or other foods such as jacket potatoes, vegetables and in cooking and baking.*</td></tr>
<tr><td>Women</td><td>No more than 3 teaspoons</td></tr>
<tr><td>Cooking Oils</td><td>Men & Women</td><td>No more than 1.5 teaspoons</td><td>You should use the oils when cooking or making salad dressings*</td></tr>
<tr><td>Cheese</td><td>Men & Women</td><td colspan="2">We do not need you to eat specific amounts of cheese but when you do eat cheese please select low fat varieties as shown in the Foods to Choose/Avoid table.</td></tr>
<tr><td>Milk</td><td>Men & Women</td><td colspan="2">Please use Skimmed milk on the cereal provided, in your tea and coffee and in any recipes including milk.*</td></tr>
<tr><td>Breakfast cereals/ Bread/Rice/Potatoes</td><td>Men & Women</td><td colspan="2">Please eat the study breakfast cereals, bread, rice and potatoes in the same quantities you eat in your usual diet plus 2 portions/day of either one or two of the foods, shown below.
i.e. Please eat _______carbohydrate portions per day

<table>
<tr><th rowspan="2">Food</th><th colspan="2"></th><th rowspan="2">Measures:</th></tr>
<tr><th>Men</th><th>Women</th></tr>
<tr><td>Breakfast cereals</td><td>6</td><td>4</td><td>heaped tablespoons</td></tr>
<tr><td>Study Bread</td><td>1½</td><td>1</td><td>medium slice</td></tr>
<tr><td>French Bread</td><td>2</td><td>1½</td><td>inch slice</td></tr>
<tr><td>Study Rice, cooked</td><td>3</td><td>2</td><td>heaped tablespoons</td></tr>
<tr><td>Potatoes</td><td>1½</td><td>1</td><td>egg-sized potato</td></tr>
<tr><td>Chapatti, wheat flour</td><td>1½</td><td>1</td><td>small (6” diameter)</td></tr>
</table>

Avoid pasta.</td></tr>
<tr><td>Snacks</td><td>Men & Women</td><td colspan="2">Please replace one snack that you normally eat during the day with one of the biscuits we provide</td></tr>
</table>

- * Please remember if you use the study fat spreads, oils, cheese and milk to make meals to be eaten not only by yourself but other members of your family/household, you will need to divide the amounts added to the recipe by the number of people consuming the meal to calculate how much **you yourself have eaten**.
- * For example if you use a tablespoon of oil in a recipe for two people including yourself you will have eaten about half a tablespoon.

Foods to Choose/Avoid

Eating at home

	Choose	Avoid
**Spreads**	Spread provided	All other types of spread
**Cooking oils**	Oils provided	All other types of cooking oil.
**Cheese**	Half-fat or reduced fat including Heinz half fat cheddar, Tesco healthy eating/living varieties, Sainsbury's be good to yourself, Asda 'Good for you' varieties, Edam & cottage cheese.	Full fat
**Milk**	Skimmed<br>Low fat yoghurt	Semi skimmed, whole and soya<br>Cream
**Breakfast cereals**	Cereals provided (Branflakes, Rice Crispies, Balance)	All other breakfast cereals
**Bread**	Bread provided or French bread. Other types of bread in smaller quantities.	
**Rice/Pasta/Potatoes**	Rice provided and potatoes	Basmati rice and all types of pasta
**Snacks**	Biscuits provided (to replace one usually eaten snack/day	

Eating out

	Foods to choose	Foods to avoid
**Spreads**		Avoid all spreads
**Cooking oils**		Avoid adding additional oils or salad dressings
**Cheese**	Low fat varieties if specified including cottage cheese & Edam.	Avoid cheese or cheese-based dishes/sauces if type is unspecified
**Milk**	Ask for skimmed milk	Whole milk, cream,<br>cream-based desserts & sauces
**Breakfast cereals**	Choose Branflakes or Rice Crispies	Avoid all other types
**Bread**	Any types of bread	
**Rice/Pasta/Potatoes**	Choose potatoes or bread as an accompaniment	Avoid dishes with pasta or basmati rice (choose naan bread rather than rice in Indian restaurants)
**Snacks**	Fruit, low fat snacks<br>Sorbet, very low-fat ice-cream	High fat snacks such as crisps, nuts, croissants, pastries.
