# Supplementary material for: SFAs do not impair endothelial function and arterial stiffness1
Source: Am J Clin Nutr. 2013 Aug 14;98(3):677–83. doi: 10.3945/ajcn.113.063644 (PMC3743730; doi:10.3945/ajcn.113.063644)
Supplement: Supplemental data [file 98.3.677_ajcn063644SupplementaryData2.doc]

**Supplementary Table 1.** Fatty acid composition of plasma phospholipids at baseline after 4 wk run-in on a saturated fatty acid rich reference diet (HS) and at 24 wk following randomization to the HS diet or diets low in SFA and high in monounsaturated fatty acids (HM) or carbohydrate (HC).

| Fatty acid | HS RUN-IN^1^ | HS^2^ | HM^3^ | HC^4^ |
| --- | --- | --- | --- | --- |
| 14:0 | 0.24 ± 0.13 | 0.23 ± 0.11 | 0.25 ± 0.12 | 0.23 ± 0.11 |
| 16:0 | 26.52 ± 3.08 | 26.55 ± 3.41 | 26.77 ± 5.18 | 26.93 ± 4.54 |
| 16:1*n*-9 | 0.51 ± 0.24 | 0.54 ± 0.24 | 0.50 ± 0.19 | 0.50 ± 0.23 |
| 18:0 | 16.01 ± 1.70 | 16.25 ± 1.98 | 16.09 ± 2.10 | 15.91 ± 1.70 |
| 18:1*trans* | 0.22 ± 0.13 | 0.18 ± 0.09 | 0.22 ± 0.11 | 0.21 ± 0.11 |
| 18:1*n*-9 | 9.66 ± 1.47 | 9.39 ± 1.21 | 10.12 ± 1.71^5^ | 9.69 ± 1.60 |
| 18:2 trans | 0.03 ± 0.03 | 0.03 ± 0.04 | 0.04 ± 0.04 | 0.04 ± 0.04 |
| 18:2*n*-6 | 20.38 ± 3.20 | 20.14 ± 2.56 | 19.88 ± 3.49 | 19.81 ± 2.69 |
| 18:3*n*-6 | 0.08 ± 0.06 | 0.08 ± 0.07 | 0.08 ± 0.07 | 0.07 ± 0.06 |
| 18:3*n*-3 | 0.24 ± 0.13 | 0.24 ± 0.16 | 0.22 ± 0.14 | 0.21 ± 0.11 |
| 20:3*n*-6 | 3.62 ± 0.98 | 3.62 ± 0.87 | 3.83 ± 1.14 | 3.71 ± 1.12 |
| 20:4*n*-6 | 10.84 ± 2.63 | 11.64 ± 2.85 | 11.38 ± 2.68 | 11.08 ± 2.36 |
| 20:5*n*-3 | 1.85 ± 1.28 | 1.73 ± 0.81 | 1.58 ± 0.88 | 1.81 ± 1.07 |
| 22:5*n*-3 | 1.30 ± 0.30 | 1.27 ± 0.21 | 1.22 ± 0.31 | 1.33 ± 0.30 |
| 22:6*n*-3 | 5.38 ± 1.97 | 5.38 ± 1.45 | 4.74 ± 1.67 | 5.38 ± 1.81 |

Mean values ± SD

^1^ n=112,^2^n=30,^3^ n=44,^4^ n=38

^5^ *P*<0.05 significantly different from SFA diet using Bonferroni’s Multiple Comparison test.
